# Functional improvement in hip pathology is related to improvement in anxiety, depression, and pain catastrophizing: an intricate link between physical and mental well-being

**DOI:** 10.1186/s12891-021-04001-5

**Published:** 2021-02-03

**Authors:** Paul Gudmundsson, Paul A. Nakonezny, Jason Lin, Rebisi Owhonda, Heather Richard, Joel Wells

**Affiliations:** 1 Department of Orthopaedic Surgery, University of Texas Southwestern Medical Center, 1801 Inwood Rd, Dallas, TX 75390 USA; 2 Department of Population and Data Sciences, Division of Biostatistics, University of Texas Southwestern Medical Center, Dallas, USA; 3 Department of Psychology, TX Scottish Rite Hospital, Dallas, USA

**Keywords:** Pain catastrophizing, Hip function, Outcomes, Mental health

## Abstract

**Background:**

Pain catastrophizing, anxiety, and depression are risk factors for poor functional outcomes and worse post-treatment pain that can be treated alongside physical care given to orthopedic patients. While these factors have been shown to be common in patients with hip pathology, there is limited literature that follows these conditions throughout treatment. The purpose of this study was to track psychological factors in patients with various hip pathology to determine if they improved alongside functional measures following treatment.

**Methods:**

Patients presenting to a specialist hip clinic were prospectively evaluated for outcomes of pain catastrophizing, anxiety, depression, and hip function. Pre- and post-treatment assessments were undertaken: Pain Catastrophizing Scale, the Hospital Anxiety Depression Scale, the Hip Outcome Survey, and Hip Disability and Osteoarthritis Outcome Score (HOOS). Patient characteristics were recorded. A correlation analysis, using the Spearman partial correlation coefficient (*r*_s_), was conducted to evaluate the relationship between change in psychological factors with change in functional outcomes.

**Results:**

A total of 201 patients (78 male, 123 female) with a mean age of 53.75 ± 18.97 years were included, with diagnoses of hip dysplasia (*n* = 35), femoroacetabular impingement (n = 35), lateral trochanteric pain syndrome (*n* = 9), osteoarthrosis (*n* = 109), and avascular necrosis of the hip (*n* = 13). Statistical analysis revealed a significant negative relationship between change in function level (as measured by HOOS ADL) and change in pain catastrophizing (*r*_*s*_ = − 0.373, *p < 0*.0001), depression (*r*_*s*_ = − 0.363, p < 0.0001), and anxiety (*r*_*s*_ = − 0.264, *p = 0*.0002). Pain catastrophizing, depression, and anxiety improved with function. Spearman correlation coefficients also revealed that pain catastrophizing, HADS anxiety, and HADS depression improved with improvement in other patient-reported functional outcomes.

**Conclusions:**

Patients with hip pathology often exhibit pain catastrophizing, anxiety, and depression, but improvements in hip functionality are associated with decreased severity of these psychological comorbidities. Exploring this connection demonstrates the correlation between musculoskeletal impairment and psychosocial outcomes and mental health. Perioperative multidisciplinary assessment may be a beneficial part of comprehensive orthopaedic hip care.

**Supplementary Information:**

The online version contains supplementary material available at 10.1186/s12891-021-04001-5.

## Background

Hip pain contributes to reductions in physical function and ability, but pain is a subjective experience that represents a confluence of biological, social, and psychological factors. The development of hip pain is multifactorial, affected by pathologic factors like hip morphology, arthrosis, and musculotendinous injury, as well as externally-related contributors like BMI, comorbid conditions, and mental health [1–5]. Pain catastrophizing, anxiety, and depression have been shown to play a role in patients’ pain and function, secondary to orthopedic conditions like osteoarthritis [2, 3, 6, 7].

Pain catastrophizing is defined as an exaggerated negative mental set that arises in response to present or anticipated pain, broken down into subcategories of *rumination*, *magnification*, and *helplessness* [8]. It is associated with patients who consistently report higher levels of perceived pain, leading to longer hospital stays and increased risk of opioid abuse following orthopedic surgery [7, 9]. Anxiety is intensified feelings of fear, worry, and nervousness [10]. Depression is a persistent and abnormal sense of sadness, hopelessness, and loss of self-worth [10]. Each plays a role in a patient’s experience of his/her hip condition and should be a part of one’s treatment. Attention to these psychological components may represent a clinically important target for the improvement of orthopedic patient health and outcomes [2].

Patients with hip pathology often exhibit levels of pain catastrophizing, anxiety, and depression at the time of presentation [11]. These psychological symptoms represent modifiable risk factors that can be treated alongside the orthopedic diagnosis. There is currently a paucity of research on how an improvement in hip function would affect psychological factors. Thus, the aim of this study was to explore the interplay between patient-reported hip function and the severity of pain catastrophizing, depression, and anxiety following orthopedic treatment.

## Methods

### Participants

We prospectively evaluated patients presenting at a single academic hip clinic, with the main complaint of hip pain, between August 2017 and November 2019. These patients underwent assessment using validated scales following Institutional Review Board approval. They included the Pain Catastrophizing Scale (PCS), the Hospital Anxiety Depression Scale (HADS), a visual analogue pain scale (VAS), the Hip Outcome Survey (HOS), and the Hip Disability and Osteoarthritis Outcome Score (HOOS) [8, 12–18]. These assessments and scales have been previously published and can be found at the above-listed citations. We followed these psychological measures longitudinally throughout physical and functional treatment of patients with symptomatic hip dysplasia (DDH), femoroacetabular impingement syndrome (FAI), lateral trochanteric pain syndrome (LTP), hip osteoarthritis (OA), and avascular necrosis of the hip (AVN). Additionally, the patients were informed that de-identified information from the surveys may be used in future research studies, and thus all of the patients included in this study provided informed consent.

All patients in the study presented with hip pain and diagnostic evaluation including: clinical and radiological examination by an orthopedic surgeon specializing in hip preservation and reconstruction. DDH was diagnosed based on physical and radiological examination with a lateral center-edge Wilberg angle < 20° [19–23]. FAI was diagnosed based on physical and radiological examination where there was evidence of acetabular over-coverage and/or decreased head and neck offset [19, 21, 24, 25]. The use of such radiographic cutoffs to classify the abnormal hip morphology of DDH and FAI have been shown to be 95% (95% CI: 93.7–96.1) and 94.0% (95% CI: 92.5–95.2) accurate, respectively [26]. LTP was diagnosed by physical examination with reproducible pain on palpation, lateral pain on the FABER (flexion abduction external rotation) test, and a normal plain radiograph, a method with mean sensitivity and specificity of 81 and 82% [27–29]. OA was characterized by a history and physical findings of pain and stiffness of the hip with radiological evidence of OA [19, 30]. Using this combination of findings to diagnose OA of the hip demonstrates sensitivity and specificity of 36.7 and 90.5% [19, 30, 31]. AVN was diagnosed by MRI and staged according to the method of Ficat, an approach with specificity 98% and a sensitivity of 91% in the differentiation of AVN from normal hips or those with non-AVN disease [32–35].

Patients received either operative or non-operative treatment with non-operative being physical therapy or medication/injection. The surgeries included in the operative treatment group consisted of a range of procedures tailored to the specific diagnosis and symptom severity of each patient. These operations included total hip arthroplasty, periacetabular and femoral osteotomy, and minimally invasive hip arthroscopy. Those patients treated with physical therapy were given specific physical therapy scripts including strengthening and range of motion exercises. Medications included anti-inflammatories like steroid injections and NSAIDs. Length of treatment was recorded.

**Fig. 1:**
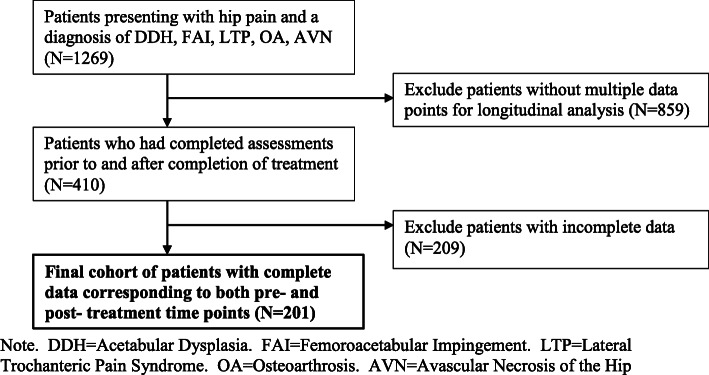
Consort Diagram of Inclusion and Exclusion Criteria. Note. DDH = Acetabular Dysplasia. FAI=Femoroacetabular Impingement. LTP = Lateral Trochanteric Pain Syndrome. OA = Osteoarthrosis. AVN = Avascular Necrosis of the Hip

The responses from 201 patients with the diagnosis of DDH, FAI, LTP, OA, or AVN were used in this study, and sample selection was primarily determined by availability of complete pre/post assessments. All patients who presented with hip pain and were ruled to have one of these five diagnoses were eligible for inclusion (*n* = 1269). These five diagnoses were chosen due to the prevalence of these conditions in the clinic’s patient population as well as clinical expertise of the senior author/diagnosing physician. However, because this aim of this study was to track functional and psychological outcomes longitudinally, only data from patients who had completed the above-mentioned assessments prior to and once after treatment were used in this study (*n* = 410). Furthermore, because all of the patient-reported data required scoring the assessments that were completed by the patients themselves, all patients with surveys deemed to be incomplete were excluded, leading to our final cohort size of 201 patients with complete pre/post data. This selection process is depicted in Fig. 1.

### Procedures and measures

Patient-level variables obtained from the patient electronic medical record (EMR) included: sex, age, body mass index (BMI, kg/m^2^), diagnosis, and treatment protocol (Table 1). The American Society of Anesthesiologists (ASA) classification of physical status was calculated using comorbidities listed in the EMR [36]. A self-report questionnaire was administered to identify co-morbidities (Table 2). Patient-perceived level of pain was also quantified using a visual analogue scale (VAS; 0 = no pain; 10 = pain as bad as it can be). The Tönnis classification was used to grade the severity of OA (Table 3) [19]. While these categories were not explicitly part of our statistical analysis of functional and psychological outcomes, they are included as descriptive statistics to better characterize our cohort.

**Table 1:** Demographic and clinical characteristics of the overall sample

Characteristic	Overall Sample (*N* = 201)
**Patient Demographics**	
Age, years, M (SD)	53.75 (18.97)
Female Gender, % (n)	61.19 (123)
**Patient Factors**	
BMI, kg/m^2^, M (SD)	27.24 (5.48)
Time pre- to post-treatment, days, M (SD)	187.36 (125.19)
*Tonnis Grade, % (n)*	
0	22.4 (45)
1	17.9 (36)
2	10.4 (21)
3	49.3 (99)
*ASA Classification, % (n)*	
1	36.8 (74)
2	46.8 (94)
3	15.4 (31)
4	1.0 (2)
History of Surgery on Current Hip, % (n)	35.8 (72)
**Diagnosis Groups**	
Femoroacetabular Impingement, % (n)	17.4 (35)
Acetabular Dysplasia, % (n)	17.4 (35)
Osteoarthrosis, % (n)	54.2 (109)
Lateral Trochanteric Pain Syndrome, % (n)	4.5 (9)
Avascular Necrosis of the Hip % (n)	6.4 (13)
**Treatment Groups**	
Surgical % (n)	83.6 (168)
Non-surgical % (n)	
Physical Therapy	11.9 (24)
Medication/Injection	4.5 (9)

Note. *M* Sample Mean, *SD* Standard Deviation. All characteristics were self-reported by the patient

*ASA* American Society of Anesthesiologists

**Table 2:** Patient Comorbidities

Patient Comorbidities	Patients, n (%)
Low Back Pain	111 (55.2)
High Blood Pressure	67 (33.3)
Cancer	21 (10.4)
Anemia	12 (6.0)
Lung Disease	9 (4.5)
Heart Disease	12 (6.0)
Liver Disease	6 (3.0)
Kidney Disease	7 (3.5)
Diabetes	12 (6.0)
Ulcer/Stomach Disease	12 (6.0)

Note. Numbers represent the number of patients with each comorbidity and the percent of our cohort that carried each comorbidity. The totals sum to a number larger than our cohort of 201 patients, as some patients carried more than one comorbidity

**Table 3:** Tönnis classification of osteoarthritis

Grade	Description	Patients, n (%)
0	No signs of osteoarthritis	45 (22.4)
1	Mild: increased sclerosis, slight narrowing of the joint space, no or slight loss of head sphericity	36 (17.9)
2	Moderate: small cysts, moderate narrowing of the joint space, moderate loss of head sphericity	21 (10.4)
3	Severe: large cysts, severe narrowing or obliteration of the joint space, severe deformity of the head	99 (49.3)

### Outcome measures

The primary outcomes were patient-reported pain catastrophizing, anxiety, depression, and hip function pre/post-treatment. Assessments were gathered using validated questionnaires completed by patients during clinic office visits both before and after treatment, with the follow-up timepoint occurring about six months on average after the completion of treatment. The PCS consists of 13 items, with scores for each question ranging from zero to four. The total score was calculated as the sum of the values of the 13 items, ranging from zero to 52. Higher scores correspond to higher levels of pain catastrophizing. A total score > 30 is considered clinically significant. HADS is a reliable patient-reported measure assessing symptoms of anxiety and depression [12, 13]. Each item score ranges from zero to three, with zero meaning no symptoms and three denoting that symptoms are felt frequently. Subscales are calculated by adding each item for a possible range of zero to 21. Each subscale score is divided into levels: normal (0 to 7), borderline abnormal (8 to 10), and abnormal (11 to 21).

Measures of patient symptoms and hip function were also included. Patient-perceived level of function was quantified using the HOS and the HOOS scores [16, 17]. The HOS includes activities of daily living (HOS-ADL) and sports (HOS-Sports) subscales. Respectively, each subscale has 17 and nine items that are scored from 0 to 4 (0 being unable to perform; 4 being no difficulty). Subscale total scores are summed and normalized so that the final scores are a percent of maximal function. The HOOS consists of 40 questions and five subscales related to stiffness (HOOS Stiffness), other symptoms (HOOS Symptoms), ADLs (HOOS ADL), function in sports and recreation (HOOS Sports), and hip-related quality of life (HOOS QOL). Each item is scored 0 to 4 (0 indicating extreme trouble and 4 meaning no trouble), and the sum is normalized so that a score of 0 indicated severe impairment and a score of 100 represents no problems.

### Statistical analysis

Demographic and clinical characteristics for the sample of 201 patients with a range of hip pathology were described using the sample mean and standard deviation for continuous variables and the frequency and percentage for categorical variables. The mean level of pain catastrophizing, anxiety, depression, and functional outcome measures at pre and post-treatment was compared using the dependent samples t-test. Next, a correlation analysis, using the Spearman partial correlation coefficient (*r*_s_), was conducted to evaluate the relationship between change in self-reported pain catastrophizing (PCS total score), HADS depression, and HADS anxiety with change in patient-reported functional outcome measures, while controlling for age, BMI, and time in days from pre- to post-treatment assessment.

Statistical analyses were carried out using SAS software, version 9.4 (SAS Institute, Inc., Cary, NC). The level of significance was set at α = 0.05 (two-tailed). We implemented the False Discovery Rate (FDR) procedure to control false positives over the multiple tests [37].

## Results

### Participant characteristics

The sample of 201 patients were 61.19% female, mean age of 53.75 ± 18.97 years (range = 14 to 89 years). Mean BMI was 27.24 ± 5.48 kg/m^2^. The surgical group included 168 patients, while the non-operative group included 33 patients (physical therapy = 24, medication/injection = 9). The mean time in days from pre- to post-treatment assessment was 187.36 ± 125.19 days (range = 28 to 725 days). The sample included 35 DDH patients, 35 with FAI, 9 with LTP, 109 with OA, and 13 with AVN. Demographic and clinical characteristics are shown in Table 1.

### Change in PCS, HADS, and functional outcomes

The dependent samples t-test revealed statistically significant improvement in mean levels of pain catastrophizing (*p* < 0.0001), anxiety (*p* < 0.0001), depression (*p* < 0.0001), and functional outcomes (*p* < 0.0001) from pre- to post-treatment for patients with adverse hip conditions (Table 4).

**Table 4:** Change in mean levels of pain catastrophizing, anxiety, depression, and function from pre- to post-treatment for patients with adverse hip conditions

	N	Pretreatment	Posttreatment	Δ_**M**_	
		M (SD)	M (SD)	M (SD)	***p***-value
**Outcome**					
PCS Total	201	15.94 (12.73)	8.26 (10.57)	−7.67 (11.70)	< 0.0001
VAS Pain	201	5.21 (2.35)	2.51 (2.50)	−2.70 (3.11)	< 0.0001
HADS Depression	201	5.42 (3.72)	3.83 (3.61)	−1.59 (3.96)	< 0.0001
HADS Anxiety	201	5.43 (3.02)	3.90 (4.07)	−1.53 (4.11)	< 0.0001
HOS Function	201	1.91 (0.68)	2.70 (0.81)	0.79 (1.03)	< 0.0001
HOOS ADL	201	46.45 (17.40)	65.23 (15.76)	18.77 (21.39)	< 0.0001
HOOS Symptoms	201	41.04 (19.88)	64.04 (17.79)	23.00 (26.12)	< 0.0001
HOOS Stiffness	201	38.95 (20.45)	58.65 (19.43)	19.70 (26.92)	< 0.0001
HOOS Sport	201	30.43 (22.94)	50.26 (25.20)	19.83 (32.51)	< 0.0001
HOOS QoL	201	20.98 (17.47)	49.18 (24.04)	28.20 (28.52)	< 0.0001

Note. *M* = Sample Mean; *SD*=Standard Deviation; Δ_M_ = Mean change in outcome. Change was operationally defined as post minus pre level. p-value (two-tailed) = Dependent samples t-test was used to test for differences in sample means from pre- to post-treatment. FDR values were all 0.0001

*PCS* Pain Catastrophizing Scale, *VAS* Visual Analogue Pain Scale, *HADS* Hospital Anxiety and Depression Scale, *HOS* Hip Outcome Survey, *HOOS* Hip Disability and Osteoarthritis Outcome Score

### Correlation between change in PCS and HADS with change in functional outcomes

The Spearman partial correlation coefficients revealed a significant negative relationship between change in level of function (measured by the HOOS ADL) and change in pain catastrophizing (*r*_*s*_ = − 0.373, *p < 0*.0001), change in HADS depression (*r*_*s*_ = − 0.363, p < 0.0001), and change in HADS anxiety (*r*_*s*_ = − 0.264, *p = 0*.0002), while controlling for age, BMI, and time in days from pre- to post-treatment assessment. Pain catastrophizing, depression, and anxiety improved as level of function (ADL) improved. Spearman correlation coefficients revealed that pain catastrophizing, HADS anxiety, and HADS depression improved with improvement in the other patient-reported functional outcomes (Table 5). Of the functional outcomes, the Spearman correlation coefficients revealed that improvement in HOOS ADL can be interpreted as having a greater magnitude of relative importance in the expected relationship with improvement in PCS Total and HADS Depression/Anxiety.

**Table 5:** Spearman correlation coefficients (r_s_) between the change in PCS and HADS with the change in Functional Outcomes

Change in Level of Function	N	r_**s**_	p-value	FDR
	**Change in PCS Total**			
HOS Function	201	−0.298	0.0001	0.0003
HOOS ADL	201	−0.373	0.0001	0.0003
HOOS Symptoms	201	−0.216	0.0022	0.0026
HOOS Stiffness	201	−0.200	0.0046	0.0046
HOOS Sport	201	−0.264	0.0002	0.0004
HOOS QoL	201	−0.254	0.0003	0.0005
	**Change in HADS Depression**			
HOS Function	201	−0.317	0.0001	0.0001
HOOS ADL	201	−0.363	0.0001	0.0001
HOOS Symptoms	201	−0.298	0.0001	0.0001
HOOS Stiffness	201	−0.221	0.0017	0.0017
HOOS Sport	201	−0.272	0.0001	0.0001
HOOS QoL	201	−0.271	0.0001	0.0001
	**Change in HADS Anxiety**			
HOS Function	201	−0.129	0.0690	0.0828
HOOS ADL	201	−0.264	0.0002	0.0012
HOOS Symptoms	201	−0.177	0.0123	0.0369
HOOS Stiffness	201	−0.155	0.0290	0.0580
HOOS Sport	201	−0.133	0.0617	0.0828
HOOS QoL	201	−0.105	0.1373	0.1373

Note. Change was operationally defined as post minus pre level. p-value = Two-tailed test on Spearman’s Rho. *FDR* False Discovery Rate, *HOS* Hip Outcome Survey, *HOOS* Hip Disability and Osteoarthritis Outcome Score, *PCS* Pain Catastrophizing Scale, *HADS* Hospital Anxiety and Depression Scale

## Discussion

The role of psychological factors, like that of pain catastrophizing, anxiety, and depression, in the presentation of orthopedic symptoms and patient-reported pain are becoming more recognized [1–3, 6, 11, 38]. Hampton et al. demonstrated that patients with hip pathology also present with levels of pain catastrophizing, anxiety, and depression. Hip pain and dysfunction may play a significant role in a patient’s psychological well-being [11]. We further explored this association between function and psychology; specifically, a link between improved activity of daily living function and improved psychological factors.

Our primary aim was to assess the relationship between functional improvement and levels of pain catastrophizing, anxiety, and depression. Hip function was measured in terms of the patients’ subjective assessment of their hips, rather than with performance-based functional metrics. In patients with differing hip pathologies, we found that patients endorsed a greater level of function after treatment, as measured by scores of HOS function and each of the HOOS subcategories. The most improved absolute measurements from pre-to-post treatment include the HOOS Sports, HOOS Symptoms and HOOS QoL subcategories; thus, patients endorsed the most functional improvement in lower stress, day-to-day activities. Additionally, patients endorsed a lower level of average pain following treatment, as measured by the VAS.

Assessment of the psychological parameters of this study began by establishing the levels of pain catastrophizing, anxiety, and depression present in our patients prior to their onset of treatment. Untreated patients presenting with hip pain were affected by clinically significant levels of each of these factors prior to treatment, regardless of their diagnosis. This link between hip pathology and mental health status has been previously documented in OA, FAI, and DDH [2, 3, 7–9, 11, 39]. Additionally, it has been previously shown that this relationship is quantifiable, as higher reported subjective functioning in hip patients is associated with lower levels of pain catastrophizing, anxiety, and depression at time of presentation [11, 38]. In our cohort, pain catastrophizing scores were particularly elevated in patients with lower function scores, as difficulties with activities of daily living, and the resulting loss of self-sufficiency, lend themselves to pain catastrophizing [8, 40].

After assessing the psychological profile of our cohort prior to treatment, we next determined how each measured psychological category changed following treatment. Psychologic improvement may not always occur following treatment, as previous studies have shown that significant levels of various psychiatric conditions can be present after treatment of orthopedic conditions [41, 42]. In a study of comorbidity progression following arthroscopic hip surgery, it is shown that psychological issues can worsen following treatment, perhaps due to dissatisfaction with the level of postoperative functional improvement [43]. There is no association that shows that treatment itself directly improves psychological metrics if the patient does not experience an improvement in level of function. Our results support that improvement in the mental health measures is associated with the effectiveness of treatment on improving hip function and overall quality of life.

Although improvement of each of the functional outcomes showed correlation with improvement in pain catastrophizing, HADS anxiety, and HADS depression, our results demonstrate that improvement in HOOS ADL has the greatest magnitude of importance in the relationship with improvement in pain catastrophizing and HADS Depression/Anxiety. Loss of ADL independence has been shown to cause large declines in mental health, as functional impairment and pain have a strong impact on daily life, causing patients to avoid situations and activities that require the use of their problematic hip [44, 45]. Conversely, it has been shown that relief from the factors that limit independence and social engagement can reduce feeling of helplessness and isolation, directly impacting feelings of depression and anxiety [46, 47]. Our results support this supposition: improved hip function and the decreased burden felt in ADL has a strong relationship with the improvement of mental health. Because activities of daily living represent such a large part of a patient’s quality of life it is likely that patients are more aware of their impairment if such activities are affected. Thus, functional improvement that reduces difficulty with ADL may have a particularly strong effect on overall wellness.

Of the psychological metrics that tended to improve alongside increased function, the pain catastrophizing total score demonstrated the most relative improvement. Pain catastrophizing is an important pain-related variable that has been adversely linked to disability and quality of life in patients with both hip and knee OA [48, 49]. As pain catastrophizing is an exaggerated negative mental state during painful experience, it is a logical conclusion that decreased pain and increased function reduce catastrophizing [8]. Our results support this link, revealing a trend of decreased PCS scores as functional assessment improves. As patients feel less hindered by their hip condition, their expectation and anticipation of associated pain and disability decreases accordingly.

There are a few limitations of this study. The cohort of patients included in this study presented to one specialist, and may not reflect all hip patients in a general population. Patients suffering from OA represented a majority of the diagnoses, and the small sample size of diagnoses like LTP and AVN prevented our ability to run a stratified analysis on each subgroup. However, because we are investigating the interplay between overall hip function and its impact on psychosocial health rather than any specific disease-modifying process, we deemed it appropriate to analyze our overall cohort. Additionally, our assessments lack standardization in both length of time from the pre- to post-treatment measurement as well as treatment protocol. Although about 85% of patients received surgery (compared to about 15% who received non-operative treatment) and we statistically controlled for length of time from pre-to-post treatment, the wide range in time elapsed between pre/post assessments and the varying treatment modalities may confound our results. However, we also note that the varying treatment modalities along with the time elapsed between assessments represent a real-life clinic setting—not an artificially-controlled setting—which bolsters the external validity of the current study.

Finally, concern regarding the validity and reliability of using self-report measures to evaluate musculoskeletal complaints has been documented in previous literature [50, 51]. We attempted to addressed this issue by using validated assessments, but our reliance on using patient-reported outcomes to determine hip functionality and psychological comorbidity could affect the internal validity of the study.

## Conclusions

In conclusion, we found that improvements in pain catastrophizing, depression, and anxiety are associated with better patient-reported hip function. Patients who originally presented with clinically significant levels of pain catastrophizing, anxiety, and depression demonstrated a decreased severity of these mental health conditions following treatment that improved their hip function. Additionally, our results show the effect that functional improvements may have on psychological factors; conversely, it may also be true that improvements in patients’ psychology and mental outlook may contribute to achieving optimal functional improvements for various musculoskeletal and orthopedic conditions. Because of the association between psychological factors and hip function, we believe that mental health factors may represent an important treatment target to consider as part of a multidisciplinary approach toward treatment of orthopedic hip conditions.

## Supplementary Information

**Additional file 1.** Pain Catastrophizing Scale (PCS).

**Additional file 2.** Hospital Anxiety and Depression Scale (HADS).

**Additional file 3.** Visual Analogue Pain Scale (VAS).

## Data Availability

The datasets generated and/or analyzed during the current study are not publicly available due concern for patient privacy but are available from the corresponding author on reasonable request.

## Abbreviations

ADL: Activities of Daily Living
AVN: Avascular Necrosis of the Hip
BMI: Body Mass Index
DDH: Developmental Dysplasia of the Hip
EMR: Electronic Medical Record
FABER: Flexion Abduction External Rotation Test
FAI: Femoroacetabular Impingement Syndrome
HADS: Hospital and Anxiety Depression Score
HOOS: Hip Disability and Osteoarthritis Outcome Score
HOS: Hip Outcome Survey
LTP: Lateral Trochanteric Pain Syndrome
MRI: Magnetic Resonance Imaging
OA: Osteoarthritis of the Hip
PCS: Pain Catastrophizing Scale
QOL: Quality of Life
VAS: Visual Analogue Pain Scale
