# Supplementary figures and images for: Functional improvement in hip pathology is related to improvement in anxiety, depression, and pain catastrophizing: an intricate link between physical and mental well-being
Source: BMC Musculoskelet Disord. 2021 Feb 3;22:133. doi: 10.1186/s12891-021-04001-5 (PMC7860171; doi:10.1186/s12891-021-04001-5)

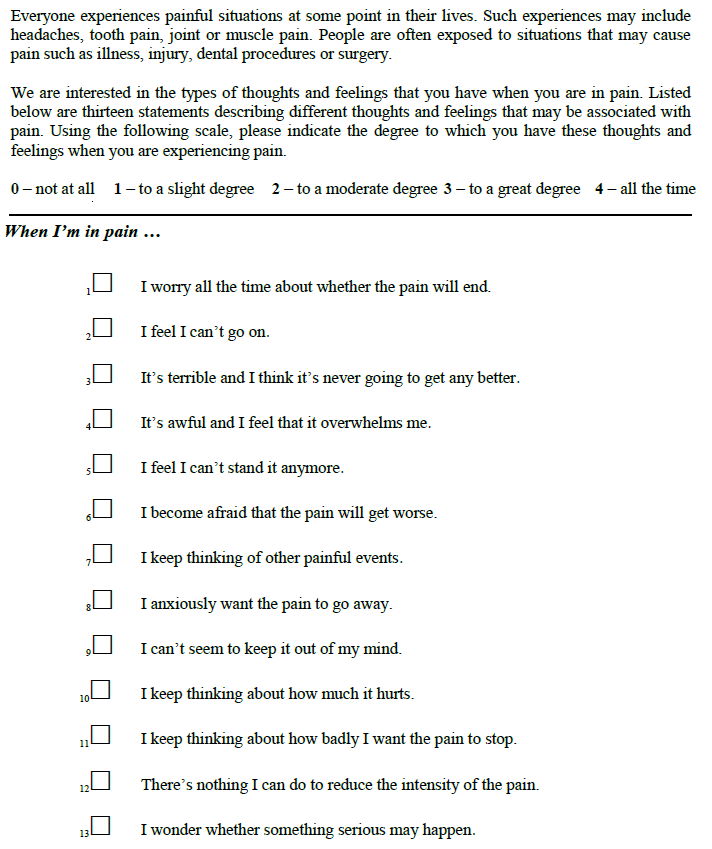

Supplement: Supplementary file 1 — Additional file 1. Pain Catastrophizing Scale (PCS). [file 12891_2021_4001_MOESM1_ESM.png]

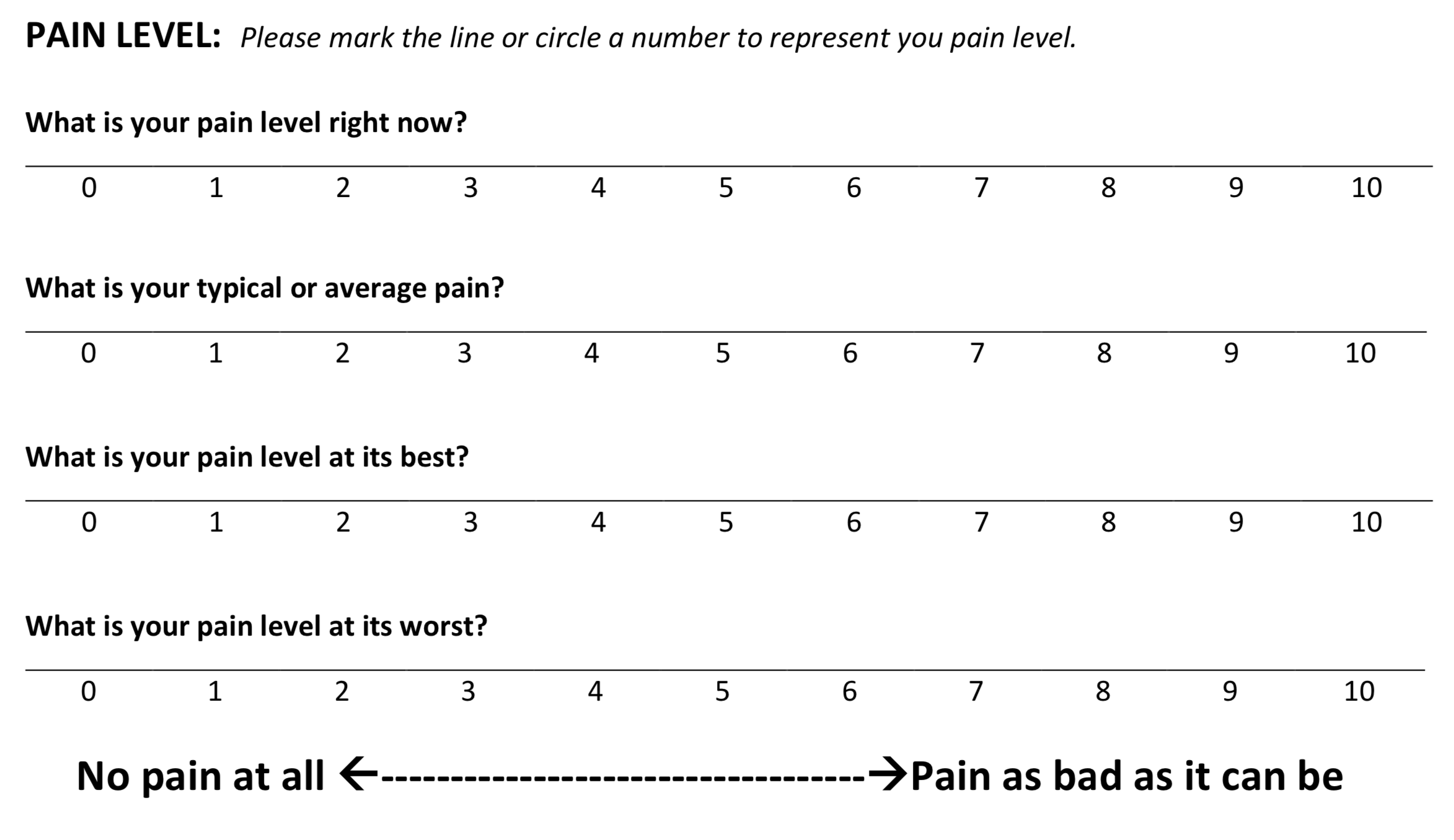

Supplement: Supplementary file 3 — Additional file 3. Visual Analogue Pain Scale (VAS). [file 12891_2021_4001_MOESM3_ESM.png]
